# On solving coordinate problems in climate model output and other geospatial datasets

**DOI:** 10.12688/openreseurope.20467.2

**Published:** 2025-12-16

**Authors:** Clément Cherblanc, Jeppe Peder Grejs Petersen, Fredrick Bunt, José Abraham Torres-Alavez, Ruth Mottram

**Affiliations:** 1 National Center for Climate Research, Danish Meteorological Institute, Copenhagen, 2100, Denmark; 2 Niels Bohr Institute, University of Copenhagen, Copenhagen, 2200, Denmark; 3 Department of Computer Sciences, University of Montana, Missoula, Montana, 59812, USA

**Keywords:** Regional climate models, Arctic, Antarctic, CDO, xarray, regridding

## Abstract

The output from Regional Climate Models (RCMs) can be difficult for non-specialists to handle, especially in cases where metadata describing coordinate systems is incomplete or absent. Standard geospatial analysis tools expect coordinate reference systems to be encoded inside file metadata. In addition to different metadata conventions, RCMs that are run over limited domains in the Arctic and Antarctic frequently have rotated longitude and latitude grids that add additional complexity compared to geographic datasets. In this article, we describe two post-processing methods that make RCM outputs easier to use for applications in the climate and related sciences. We demonstrate two different approaches that allow output from RCMs to be 1) read on the correct grid without interpolating or reprojecting the dataset, or 2) resampled onto a regular grid that includes geographic coordinates. These two approaches use the widely available and free software tools Python and Climate Data Operators (CDO). These transformations make outputs simple to use in Geographic Information Systems (GIS) and allow the full use of Python libraries, such as xarray, for plotting and analysis.

## Plain language summary

The datasets exist on grids with coordinates. The coordinates of one grid can be related to those of another using a coordinate reference system. Lack or poor encoding of coordinate reference systems can lead to incompatibilities, errors, approximations, and bottlenecks. We present ways to read the coordinate reference systems correctly and to recover them if they are missing, using two programming languages. We provide real case examples and discuss the implications of the presented methods.

## 1 Introduction

Geospatial data requires common reference frameworks to identify spatially distributed information. A significant fraction of climate science relies on large model datasets that contain geospatially and temporally varying data. A simplified approach is usually used to populate the data on grids that have geographic coordinates. In the case of geospatial data, grid coordinates must be precisely located to simplify the data analysis. Therefore, the metadata in a geospatial data file requires a Coordinate Reference System (CRS). CRS is metadata that contains information including the origin of the grid, the spacing of grid points, and the unit vectors that compose it, whether it is a Euclidean reference system or not, and other parameters. Incorrect or poorly defined CRS may lead to errors, including incorrect comparison of datasets with different locations, spatial areas not being correctly defined, and difficulties comparing point measurements with gridded outputs. The correct CRS allows for translation between grids and enables easy reading and editing of the data at the correct location.

Despite many years of working with RCM outputs, many users still have difficulties dealing with spatial outputs from climate models owing to unfamiliarity with different grid conventions and sometimes missing metadata in file headers. Large collaborations between scientists can also bring in different standards and habits, occasionally resulting in unusual data collection. Common issues also include legacy model output, and the inclusion of non-compliant datasets and ad hoc simulations that are not prost-processed to be fully CF-compliant. However, when modelling community consensus is reached, for example in CORDEX (Co-Ordinated Regional Downscaling EXperiment simulations ^1^, a single common structure is usually specified as part of the experimental protocol. Moreover, in projects outside of community-determined RCM experiments, other approaches may take different decisions regarding structuring datasets to reduce data storage requirements. This results in various data structures in output files for different models, and post-processing for analysis therefore requires additional processing to reach standardized data conventions and a consistent approach.

Some of the differences between files that we observed include, for example, the CRS being encoded in the first time-step output and not in the subsequent ones to reduce data storage, a missing CRS altogether, and the CRS not containing all the metadata required for analysis. Even when there is no discontinuity in the approach of scientists and engineers, the reading and handling of climate datasets remain complicated. Indeed, in the case of Regional Climate Models (RCM), the grid used by the model may not be on any specific CRS to simplify the model computation. The output of these RCMs outside community-determined protocols is often published raw to reduce bias introduced by regridding, with the intention that it can then be reprojected and/or resampled by users for specific use cases. High coordinate distorsion near the poles also implies that default reprojection techniques, usually to a “regular grid” (defined further), may introduce unwanted biases.

Although some sophisticated tools already exist to simplify the handling of climate model datasets 2, 3), these are rarely aimed at casual users of the climate model outputs. The output of geospatial data from RCMs is usually in the NetCDF format ^4^, and there are now widely used conventions ( https://cfconventions.org/, ^5^) that have allowed the development of tools to easily write and read such files. But CF-compliancy is not always present in legacy, preliminary output, or in other types of datasets. Having examples of multiple approaches to reading and analyzing these datasets is a key part of this research.

In this paper, we present example-based best-practices from two languages, one based on Python code and one based on the Climate Data Operators (CDO) ^6^ libraries to read, plot, interpolate, and reproject the RCM output. In the first case, we relied on xarray ^7^, rioxarray ^8^, scipy ^9^, and pyproj ^10^ Python libraries. In the second approach, we used the CDO software suite ^6^, which also provides bindings for programming languages, such as Python, Ruby, and R. This is both targeted at educating users to modern methods, and provide practical examples of the inclusion of datasets otherwise unusable. Although we provide code examples that are generalizable, we urge the reader to read the documentation of functions before use. A GitHub repository accompanies this paper, with sample datasets and Jupyter notebooks, so that the reader can test the methods described in this paper. See section “Code Availability”.

## 2 Processing climate model metadata

In this study, we primarily use the output from the Harmonie-CLIMate (HCLIM) RCM. HCLIM ^11^ is an RCM widely used in many different domains, including the Arctic and Antarctic. The model includes both hydrostatic and non-hydrostatic physical schemes and can be run at a wide range of resolutions from the mesoscale to the convection-permitting sub-kilometer scale. In this study, to introduce the methods, we used outputs at 11 km resolution from a series of simulations developed in the PolarRES project ^12^. The Python-based method was demonstrated on Antarctic simulation output from HCLIM, run at the Danish Meteorological Institute, while the CDO-based method was demonstrated on a pan-arctic simulation output run at the Norwegian Meteorological Institute.

As comparing two or more RCMs with each other and/or with in-situ observations is a common task, we also use RCM output from Arctic simulations from the MAR 3.13 climate model ^13^ and run within the PolarRES project. The MAR 3.13 model was also run on a polar stereographic grid, but at a slightly lower resolution of 12.5 km. Both HCLIM and MAR data are available in a CORDEX-compliant post-processed form ^14^, but to assess the performance of the tools developed here on unformatted output, we use the raw output before the Climate Model Output Rewriter ^15^ is applied, which, along with other operations, also applies appropriate metadata in the output NetCDF file headers.

### 2.1 Coordinate Reference System

A coordinate reference system simply consists of metadata with information on the position of a point in space and/or time. CRS can be expressed in geographic coordinates or projected coordinates. A geographic CRS uses latitude and longitude for its coordinates, usually in degrees, and functions globally. The most common geographic CRS is WGS84 or EPSG:4326 ( https://epsg.io/4326). A projected CRS uses the distance for northing and easting, usually in meters from a local point of origin, by projecting the curved Earth’s surface onto a 2D plane. Projection onto the 2D plane creates distortions that increase radially. Geographic coordinate systems work well for data that span the whole globe, whereas projected coordinate reference systems conserve distances and areas in two dimensions more closely for a given region, but they function much better locally than over larger areas.

### 2.2 Types of grids

In this study, we focused on horizontal spatial dimensions. We introduced two types of grids with their respective properties and issues.

**1. Regular grids** are defined by two linearly spaced coordinate vectors, *x* and *y*. The Cartesian product of the two vectors yields unique coordinates for each grid point. *x* and *y* commonly have units of meters or degrees (longitude and latitude, respectively).

**2. Non-regular grids** that cannot be defined by the two vectors *x*, *y* require matrices of coordinates *X* and *Y*. These grids usually require reprojecting the data onto a regular grid to handle more complex coordinates.

It should be noted that any grid can be made regular by simply using *x* and *y* indices as coordinates. However, in practice, if there is no CRS that maps these index coordinates to real-world locations, the grid is nonregular. We are concerned with georeferenced data for this study; therefore, we ignore this case.

Only regular grids can be encoded in the metadata of a NetCDF file as the dataset dimensions. Often, the outer *x* and *y* (or rlon/rlat, where r stands for rotated) vectors are stored as dimensions, but a full grid of *X* and *Y* (or longitude and latitude) is stored as variables. In both cases, CRS may be present. If there is no CRS encoded, users of data in geographical information systems (GIS) or other types of geospatial software will likely find that the CRS Well Known Text ( *CRS_WKT*, e.g. 16) fails to load when loading the file into the system.

### 2.3 Loading the CRS

In this section, we provide an example to clarify the procedure for appending CRS to a raw file. The raw output we show is from the HCLIM model ^11^ over Antarctica with no encoded CRS or WKT. We imported a NetCDF file with the following commands, which used the rioxarray Python library ^17^:


>>> import rioxarray as rxr # to use the rio functions on xarray objects
>>> import xarray as xr
>>> ds = xr.open_dataset('file1.nc')
>>> ds
< xarray.Dataset >
Dimensions : ( y : 637 , x : 739 , time : 745)
Coordinates :
    * y ( y ) float64 0.0 1.1 e +04 2.2 e +04 ... 6.985 e +06 6.996 e +06
    * x ( x ) float64 0.0 1.1 e +04 2.2 e +04 ... 8.107 e +06 8.118 e +06
    * time ( time ) datetime64 [ ns ] 1999 -01 -01 ... 1999 -02 -01
      lon (y , x ) float64 ...
      lat (y , x ) float64 ...
Data variables :
      Polar_Stereographic | S1 ...
      ts ( time , y , x ) float32 ...
Attributes :
      Conventions : CF -1.4
      institute_id : HCLIMcom
      model_id : HCLIM
      experiment_id : ANT11_eval_ERA5
      domain : ANT11
      frequency : 1 hr
      driving_model_id : ERA5
      creation_date : Sun Mar 31 03:23:30 2024
      title : Surface Temperature
      comment : Created with gl / xtool


Note that the output above can be displayed in the command line with ncdump -h file.nc. There should be a visible CRS input with


>>> print(f"Dataset CRS : { ds.rio.crs }")
Dataset CRS : None


As the CRS is not directly encoded, the information from the CRS is usually stored in Well-Known Text (WKT) ^16^ or in a variable. Retrieving the CRS can easily be performed with the argument decode_coords="all" when loading the dataset. If this is used, we obtain the following output:


>>> ds = xr.open_dataset('file1.nc', decode_coords="all")
>>> print(f"Dataset CRS : {ds.rio.crs}")
  Dataset CRS : PROJCS["undefined", GEOGCS["undefined", DATUM["undefined", SPHEROID["undefined", 6371229 ,0]] , PRIMEM["Greenwich", 0, AUTHORITY["EPSG","8901"]], UNIT["degree", 0.0174532925199433]], PROJECTION["Polar_Stereographic"], PARAMETER["latitude_of_origin", -90], PARAMETER["central_meridian", 20], PARAMETER["false_easting", 4416613.42832607], PARAMETER["false_northing", 3071812.91203247], UNIT["metre", 1, AUTHORITY["EPSG", "9001"]], AXIS ["Easting", EAST], AXIS["Northing", NORTH  ]]


For this specific file, a *custom* CRS is available because decode_coords="all" caused xarray to decode the in- formation stored in the Polar_Stereographic variable. The methods presented here work consistently regardless of the presence or absence of this information. Here, the created CRS contains all the information required to reproject the dataset, including the projection method, parameters, units, and axes. This dataset can now access the functionalities of xarray or the powerful reprojection functions of rioxarray, and can be exported in a format readable by GIS tools. Although the rioxarray reproject function is powerful, it will reproject the dataset to a regular grid. If the data was not a regular grid in the first place, there may be errors in the reprojection, and reprojecting the whole dataset to a regular grid may anyway not be the intended outcome. Transforming only the coordinates will alleviate those limitations.

## 3 Transformation of coordinates

**Figure 1.**
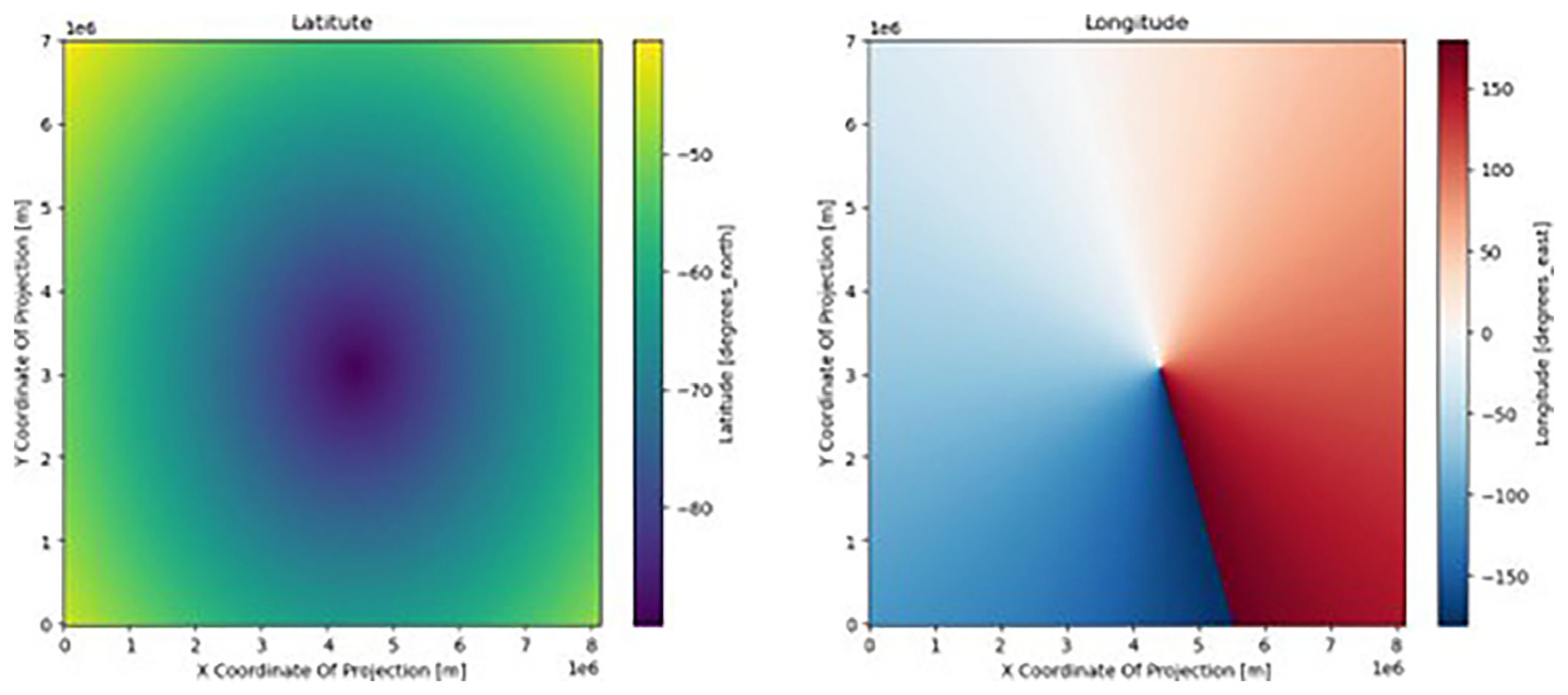
Visualizing the latitudes (left) and longitudes (right) fields. The x and y axis are the default x and y coordinates (in meters) of the dataset, inherited from the creator of the dataset; this grid has no CRS and no connection to the real world.

In files where CRS and WKT are missing or corrupted, information on the coordinates can usually be retrieved from other fields of the dataset. Most of the datasets have secondary coordinate grids that contain useful information. In the above example, the *lat* and *lon* grids are in degrees of latitude and longitude, which are commonly assumed to be EPSG:4326, the most recent and widely used global geographic CRS ^18^. On rare occasions, this information is encoded only in the first time step of the model output. In our case, irregular grid information is present in all files, and offers an alternative to the decoding of the dataset CRS. First, we plot *lat* and *lon* ( Figure 1) and find that they are irregular grids ( Section 2.2). The dataset is in a polar stereographic projection, so *lat* increases radially, whereas *lon* increases angularly about the pole.

A new grid of coordinates within any CRS can be obtained by transforming the lat/lon grids. CRS EPSG:3031 is a projected CRS centered around the South Pole and is often used for data from Antarctica. We know that this CRS is appropriate for this work, but the CRS will differ for other regions. Latitude and longitude should always be EPSG:4326, but there is no global standard for projected CRS because it is region-dependent. If no recommendation is formulated for a dataset, and there is no standard for the region of study, the Universal Transverse Mercator (UTM) system should be used. UTM provides a grid of local CRS, where distortions from reprojection are limited to a minimum, but grow away from the grid origin. The appropriate UTM CRS for a given study site can be estimated with the estimate_utm_crs function of rioxarray. The pyproj library offers transformers that achieve the transformation


>>> from pyproj import Transformer
>>> transformer = Transformer.from_crs(4326 , 3031 , always_xy = True )
>>> transformer
< Concatenated Operation Transformer : pipeline >
Description : axis order change (2 D ) + Antarctic Polar Stereographic
Area of Use :
- name : World
- bounds : ( -180.0 , -90.0 , 180.0 , 90.0)


**Figure 2.**
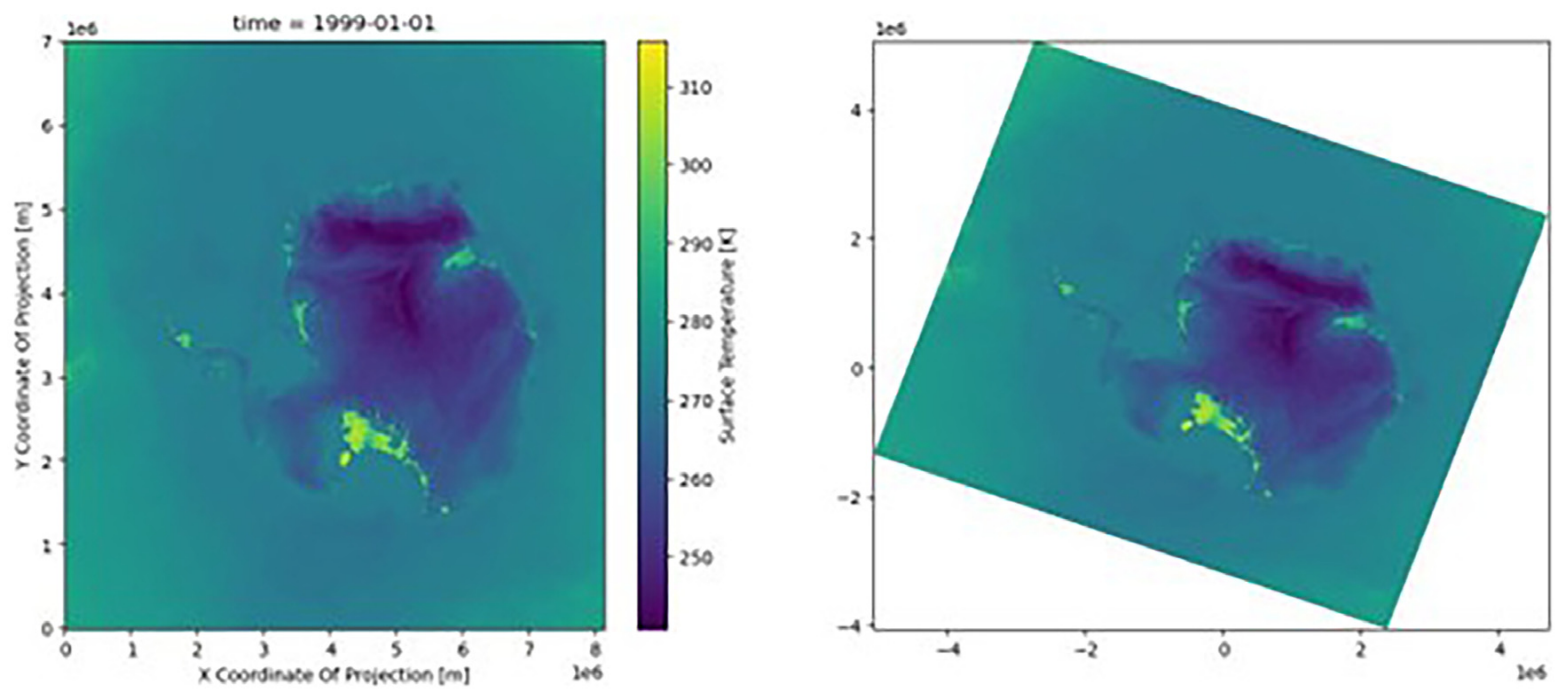
Plot of the dataset (skin temperature) with the native coordinates (left) and the new reprojected coordinates (right) with CRS. The color scale was identical in the two figures; the data were not affected, only the location at which the data were presented.


The transformer can be applied to the dataset’s latitude and longitude arrays, producing Figure 2.

>>> x_reprojected, y_reprojected = transformer.transform(ds.lon, ds.lat)
>>> import matplotlib.pyplot as plt
>>> fig , axs = plt.subplots(ncols =2, figsize =(15,6))
>>> ds.ts[0].plot(ax = axs[0])
>>> axs[1].pcolormesh(x_reprojected, y_reprojected, ds.ts[0])


*x_reprojected* and *y_reprojected* are now in a known CRS, but are still 2D coordinate matrices. However, we still do not have a regular grid.

## 4 Reprojecting the whole dataset

For spatial comparisons and analysis of two different datasets, both must be on the same grid such that the matching cell values can be associated with each other. Reprojecting the dataset implies altering the data values via interpolation, which must be performed carefully to avoid introducing artificial biases. We reproject the variable var of the dataset source_ds onto the grid of the target_ds.nc dataset. Note that the two datasets may have geographic or projected CRS, but the Python and CDO approaches diverge in how they handle them. In both cases, however, the two datasets must span the same region with the same variable, unit, and time resolution, thus making a physical sense to compare them.

### 4.1 Reprojection using Python-based method

In Python, two datasets with a CRS can be reprojected using the source_ds.rio.reproject_match(target_ds) function. If the CRS is not properly encoded, manual reprojection can be reduced using the scipy’s griddata function ^9^.

Because the geographic coordinates are spherical, the boundary is periodic; therefore, we use the projected CRS.


>>> from scipy.interpolate import griddata
>>> transformer = Transformer.from_crs(4326, 3031, always_xy=True) # same as above , you may need to use two transformers for the two different datasets
>>> x_source, y_source = transformer.transform(source_ds.lon, source_ds.lat)
>>> x_target, y_target = transformer.transform(target_ds.lon, target_ds.lat) # differs for other CRS
>>> new_data = griddata(
                  list( zip( x_source.ravel(), y_source.ravel())),
                  source_ds.var[0].data.ravel(), # first time instance of the variable
                  list(zip(x_target.ravel(), y_target.ravel())),
                  method = "cubic", # or another interpolation method
               )


### 4.2 CDO-based method

In this section, we demonstrate a different method for handling geospatial data by using the CDO package. In this example, two model simulations run on different grids need to be compared to quantify their differences. We use the HCLIM model run for the Arctic as our base grid and reproject the output from the MAR regional climate model onto the same grid. Both models were run in polar stereographic coordinates, but the MAR model grid had a slightly different resolution and shape than HCLIM. The details of these experiments are provided in 12. When using the CDO, additional steps to ensure the correct format of the data are useful. For example, extra coordinate projections may be encoded as variables in a NetCDF file, causing the output files to have numerous unused variables. A safe approach is to use the selvar function to extract only the desired variable, var, in our case:


cdo selvar,var source_ds.nc source_ds_var.nc


For CDO to reproject accurately, there must be a description of the reprojected grid in place. This is known as the target grid. In this case, it is the standard model grid from the HCLIM and is created as follows:


cdo griddes target_ds.nc > target_grid.txt
    cdo    griddes: Processed 1 variable [1.00s 62MB].


CDO uses gridded weighting for the interpolation process; it is not strictly necessary to write out a separate weights file, but if multiple files are to be reprojected, then generating a separate weights file will speed up the projection workflow significantly; otherwise, each file will need to generate weights first before interpolating. A weight file was created for remapping with a bilinear interpolation as an example (the choice of interpolation method will depend on the end-user’s aims and is not discussed here) using


cdo genbil,target_grid.txt source_ds_var.nc weights.nc
    cdo    genbil: Bilinear weights from curvilinear (646x650) to curvilinear (629x709) grid 
    cdo    genbil: Processed 1 variable over 1 timestep [2.01s 192MB]


The weight file generated with CDO can now be used to reproject any file from the same original grid to the target grid, including for different time periods. As creating weights is a computationally demanding task, reusing the weights file generated in this manner is recommended.


cdo remap,target_grid.txt,weights.nc source_ds_var.nc source_ds_on_target_grid.nc 
    cdo    remap: Processed 1 variable over 1 timestep [1.19s 177MB].


**Figure 3.**
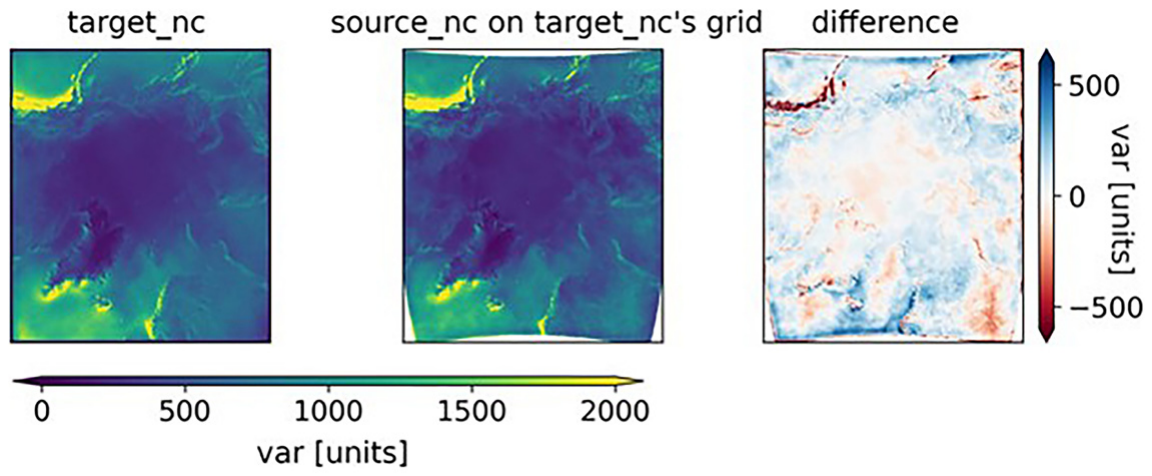
From left to right: the target_nc dataset, the source_nc dataset that was reprojected onto the target_nc grid, and the difference between the two. In the rightmost plot, the large anomalies in coastal regions with mountains (Norway and Canada) suggest that the models have a different way of calculating var in regions with rapidly changing topographies.

Figure 3 compares the data on the reprojected grid (center subfigure) with the output data from the original model. The distortion due to the models not being run in exactly the same region can be clearly seen in the anomaly subplot on the right.

The history of operations performed on a NetCDF file is usually written by CDO into the file header by default, unless suppressed to reduce file sizes. Reprojection is relatively common and may cause difficulties in reading and/or reformatting files. Therefore, we recommend that unless other considerations such as file size are important, projection information and history of reprojections should be stored as a standard in the netdf metadata. This includes the history of the dataset and commands used to build the current files. This is particularly helpful when identifying whether mistakes or incomplete information have resulted in errors in the interpretation of the NetCDF file. For example, a variable may be multiplied by a constant to change the units, but the unit field may not be updated in the NetCDF metadata.

## 5 Reprojecting onto a regular grid

**Figure 4.**
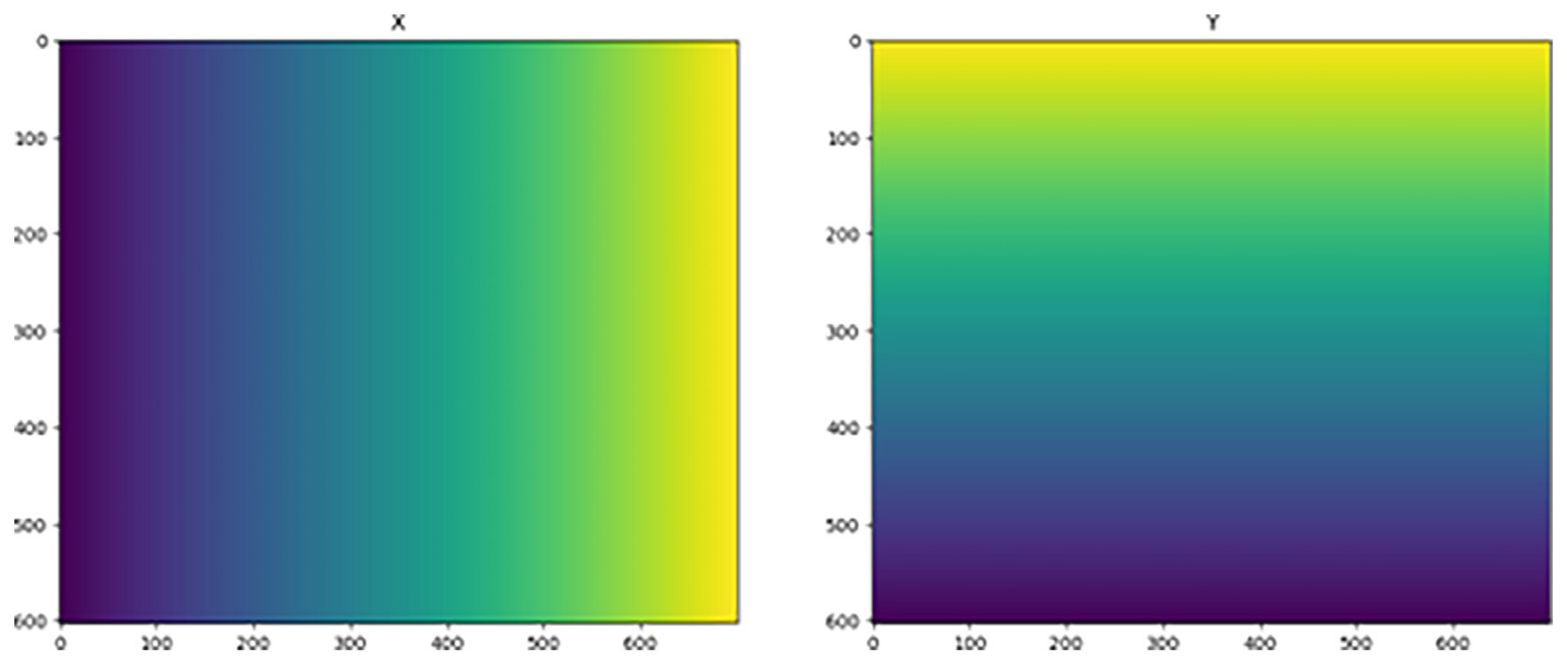
Target grids X (left) and Y (right).


Reprojecting on a regular grid implies that the dataset can then be saved with the CRS input as a new NetCDF. The coordinates of any dataset can be multidimensional, such as lat and lon in the examples above, but these multi-dimensional coordinates cannot be set as the dataset dimensions of the xarray dataset object. Only 1-dimensional vectors can be used here, because they are components of a regular grid. In the examples above, the data values are not on a regular grid, and the only way to attain this is to interpolate the data onto the desired regular grid. Data interpolation must be performed with careful consideration of the end goal. Specifically, it should be clear that a published and shared geospatial dataset has (or has not) been reprojected to avoid the introduction of interpolation artifacts. Generally, it is recommended to use the least transformed dataset to minimize the risk of errors. The reprojection of our example dataset to a regular grid was very similar to that presented in Section 4.1, with the only difference being that our target grid was regular ( Figure 4). For a regular grid, if available, the built-in functions of rioxarray can be used, but they offer limited control over the destination grid. Instead, a full control can be achieved with a variant of the following. It is created such that the new grid resembles the old one using the aspect variable:

>>> import numpy as np
>>> aspect = ds.y.size / ds.x.size # or rlat / rlon
>>> nx = 700
>>> ny = int(aspect*nx)
>>> shape = (1 , ny , nx ) # the first dimension is for time
>>> print(f"Target Grid Shape : {shape}")
Target Grid Shape : (1 , 603 , 700)
>>> x_target = np.linspace(x_reprojected.min(), x_reprojected.max(), nx)
>>> y_target = np.linspace(y_reprojected.min(), y_reprojected.max(), ny)[:: -1]
>>> X_target , Y_target = np.meshgrid(x_target, y_target)
>>> fig, axs = plt.subplots(ncols=2, figsize=(15, 6))
>>> axs[0].imshow(X_target)
>>> axs[0].set_title("X")
>>> axs[1].imshow(Y_target)
>>> axs[1].set_title("Y")


**Figure 5.**
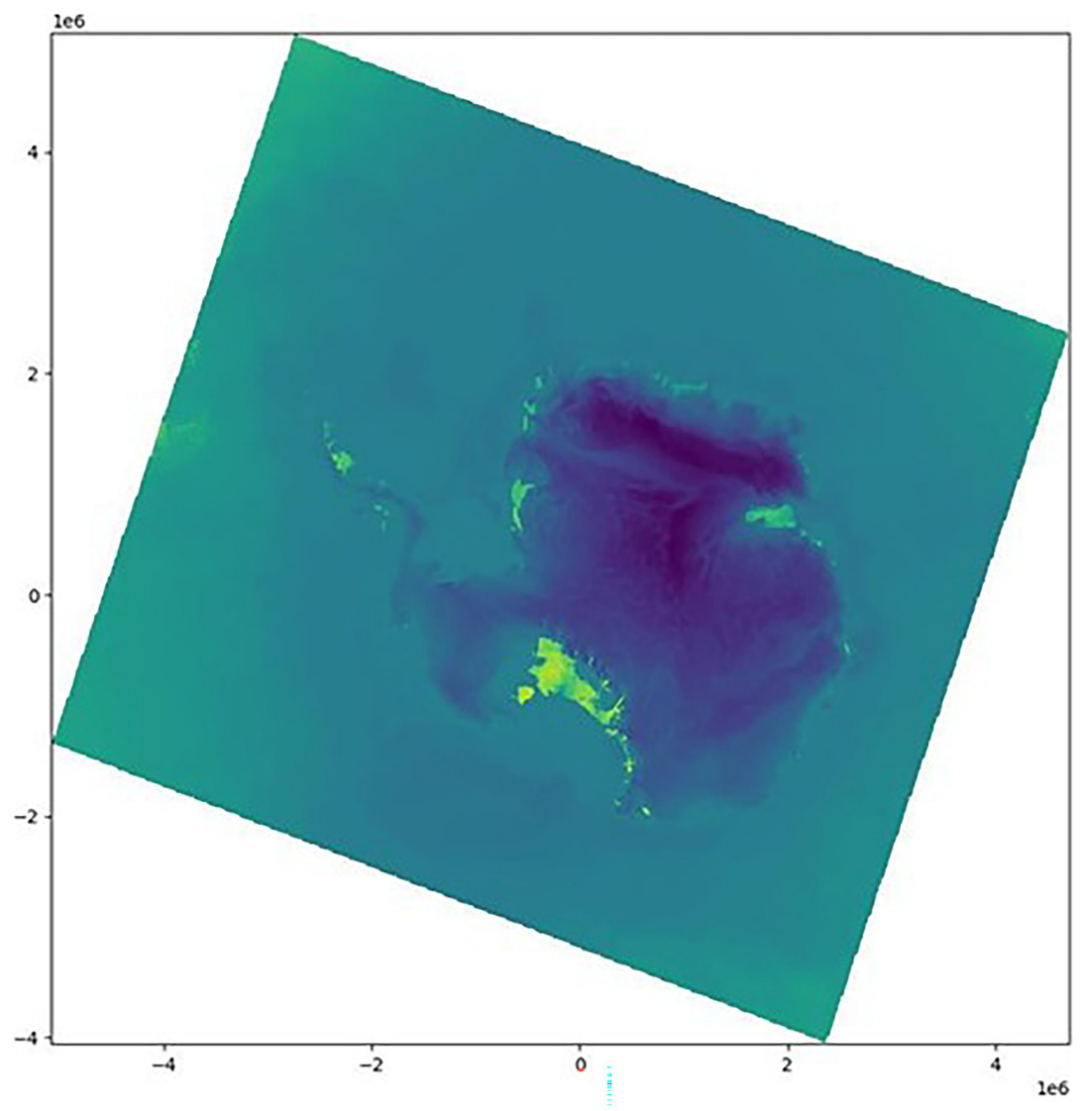
Original dataset reprojected onto a regular grid.


The reprojection can be performed using the following code and is plotted in Figure 5.

>>> regridded_data = griddata(
        list(zip(x_reprojected.ravel(), y_reprojected.ravel())),
        ds.ts[0].to_numpy().ravel(),
        list(zip(X_target.ravel(), Y_target.ravel())),
        method = "cubic"
)
>>> regridded_data = regridded_data.reshape(shape)
>>> plt.figure(figsize=(10, 10))
>>> plt.pcolormesh(X_target, Y_target, regridded_data[0])


This new dataset, with a CRS, can be saved into a new xarray object using the following code:


>>> xdata = xr.DataArray(
        regridded_data,
        dims=("time", "y", "x"),
        coords=(ds.time[:1], y_target, x_target)
).rio.write_crs("EPSG:3031")


## 6 Attaching lat/lon coordinates

We relied on the presence of additional latitude and longitude grids, but they may not always be present in the first place. Often, a separate file containing lat/lon is provided along with the model output. We provide an example from the RCM MAR ^19^, which contains SMB from 2013 (target_ds.nc). The data are only given on the model grid and lack an actual lat and lon variable, which are located in the supplementary file source_ds.nc. First, the LAT and LON variables were extracted.


cdo selvar,LAT source_ds.nc LAT.nc 
cdo selvar,LON source_ds.nc LON.nc


The LAT and LON coordinates are then merged with the target file.


cdo merge target_ds.nc LAT.nc LON.nc target_LL.nc


Finally, the LAT and LON were defined as coordinates.


ncatted -O -a coordinates,SMB,a,c,"LON LAT" target_LL.nc target_ready.nc


We now have a file similar to the first example ( Section 2.3).

## 7 Discussion

Computational efficiency is important when handling large datasets and different purposes may require different techniques. In many cases, reprojections that need to be applied to multiple files will be significantly faster using CDO, owing to its stand-alone weight computation and to not loading files to memory. We report two orders of magnitude increase in time taken from CDO to python, for the reprojection of 31 time instances of a 739 x 637 grid to a 498 x 498. For other applications in which the reprojection of coordinates is sufficient, there is no significant difference in performance (around 1 to 3 second for the same 739 x 637 grid).

Not all of the techniques presented here use the full potential of the rioxarray’s functions and NetCDF’s properties. These libraries are optimized and contain numerous functions, many of which have been optimized and rely on common operators and other libraries. The xarray library builds upon numpy and pandas ^20^ and is complemented by rioxarray that enhances the CRS and reprojection functionalities of xarray and introduces other libraries indirectly, such as pyproj and GDAL ^21^. These functions work properly on a dataset with a regular grid, but for datasets that are not on a regular grid, the operations detailed here will assist in analysis. Even so, we emphasize that users should apply data interpolation carefully, as it may act to adjust data in a non-physical way, obscuring important features in the analysis. A full description of these errors is beyond the scope of this paper, but, for example, 22 provides a full overview of the complications introduced by interpolation of both RCM and observational datasets. Even so, the model resolution may not capture physical processes at the correct scale, and interpolation may exacerbate this. Despite these drawbacks, interpolation may be the best way to answer certain questions, and the procedures we introduce here will not only assist in visualizing data, but also in reprojecting and choosing appropriate interpolation schemes.

## Conclusion

Geospatial datasets are tensors of data, and each value has spatial and temporal coordinates. Spatial coordinates are usually geographically referenced, meaning that a Coordinate Reference System (CRS) must complement the spatial coordinates to locate the grid on Earth and with respect to another grid. A CRS is usually present in a climate model dataset unless it has been neglected in the output and post-processing of data, or if the grid is not regular. Irregular grids cannot be decomposed into two vectors of x and y (or lat and lon) coordinates, only into two matrices of those coordinates. In this paper, we describe two methods that outline how to apply a CRS or bypass the lack of CRS using different methods, techniques, and libraries. Choosing between these methods is very much dependent on a user’s aims and needs. The first method consists of reprojecting the coordinates without altering the data using the pyproj transformer function. This method is fast, does not alter the data through interpolation, and allows for numerous simple computations. However, it does not allow the use of xarray and rioxarray functions because it does not create a new dataset and therefore may limit subsequent analyses. The second method creates a new dataset by interpolating the data onto a regular grid with a CRS, using the scipy’s griddata function or CDO. The outcome is a new dataset that can be saved and loaded, on which all of the libraries’ advanced and optimized functions can be used. The choice between reprojecting the coordinates only or the entire dataset depends on the user’s goals and applications. Interpolating the data introduces uncertainties and potential errors; however, it may be necessary to compare or combine two datasets of different origins. Only reprojecting the coordinates reduces contamination of the data but limits the use of certain functions. Nonetheless, methods exist to properly read, process, and reproject datasets for a multitude of purposes, and our description here is intended to assist non-modelling specialists in displaying, visualizing, and analyzing climate model data.

## Ethics and consent statement

*Ethical approval and consent were not required*

## Data Availability

The datasets are available at Zenodo under the CC-BY license, at the link: https://doi.org/10.5281/zenodo.15114614 ^23^.

## Code availability

A supplementary Github repository with Jupyter notebooks, Python code, and data to try the cdo methods can be found at https://github.com/HCLIMcom/RCM-transform. The code is available under the OSI-0BSD license.
