# Supplementary material for: On solving coordinate problems in climate model output and other geospatial datasets
Source: Open Res Eur. 2025 Dec 16;5:269. Originally published 2025 Sep 4. [Version 2] doi: 10.12688/openreseurope.20467.2 (PMC12770884; doi:10.12688/openreseurope.20467.2)
Supplement: Peer review history

# Reviewer report: Reviewer response for version 2

**DOI:** 10.21956/openreseurope.23914.r67889

**Published:** 2026-02-06

**Authors:** Mika Rantanen, Juha-Pekka Puska

**Affiliations:** 1 Finnish Meteorological Institute, Helsinki, Helsinki, Finland

**Review of Open Research Europe manuscript “On solving coordinate problems in climate model output and other geospatial datasets” by Clément Cherblanc et al.**

*This review has been conducted with a co-review with Juha-Pekka Puska, Finnish Meteorological Institute.*

 Climate modelling is increasingly going towards higher resolution, and regional climate model simulations and regional reanalyses are becoming more common. Their finer resolution offers important advantages compared to global models and reanalyses but also introduces challenges as regional model data is often not at regular lat-lon grids which is the most typical in global datasets.

 This paper provides a useful and well-structured documentation of how data on non-regular grids can (1) be read on their native grid and (2) transformed between different data projections. The authors illustrate the workflow using simple and clear examples with Python and CDO, which are (likely) the most widely used tools for this type of analysis in the climate science community.

 The manuscript has already undergone one round of peer review, and this is probably reflected in the overall quality of the presentation. The paper is clearly written, and the English is fluent. We did not identify any language-related issues. Overall, the manuscript appears to be close to publication readiness from a technical and presentation perspective.

 The only remaining concerns relate to the 1) novelty of the study and its suitability as a scientific article, and 2) the title and how well it reflects the contents of the manuscript. These comments are explained below:

 1. The paper describes established and widely used methods and, as such, does not seem to introduce a clearly identifiable methodological or conceptual novelty. Therefore, to some extent, the manuscript reads more like a technical report or a tool documentation than a scientific paper. We are not sure if this is an issue for this journal, and thus, we leave it for the editor to solve. 

 2. The title “On solving coordinate problems in climate model output and other geospatial datasets” implies a quite general treatment of geospatial data processing and their coordinate problems. However, the manuscript itself treats specific cases that are easily handled by standard tools (e.g. using the ‘decode_coords=all’ option in Xarray) or the case where a missing CRS can be easily inferred (sections 3 and 4). We thus feel that the authors should either expand the manuscript to cover a wider range of possible output formats and potential issues regarding them, or alternatively change the title to one with a narrow scope. Having a title that is not too general is important, as it makes the publication easier to discover for those that are looking to solve the specific issues addressed in the study.

 Line-to-line comments:

 Plain language summary: ‘Datasets’ instead of ‘The datasets’

 Figures are of low quality.

 Section 1 paragraph 1: A reference to a more detailed description of coordinate reference systems would be useful here on in section 2.1.

 Typo in the 2nd paragraph of Section 1 “prost-processed”.

 Some citations are written with superscript and some with normal numbers. Please check consistent use of citations.

 Section 2 paragraph 2: It is stated ‘model was also run on a polar stereographic grid’ seems to imply that the HCLIM is run on the same grid, but this is not mentioned. 

 Section 2.1 The sentence starting with “Geographic coordinate systems work well...” was hard to understand. Please rephrase. 

 Section 2.1: An example of a projected CRS could be included.

 Section 2.2: A visualization of a regular and non-regular grid could be useful for readers not familiar with them.

 Section 2.2, paragraph 5: NetCDF defines “dimension” as an integer specifying the length of a coordinate vector. The usage of “dimension” in the first two sentences contradicts this. At least in the second sentence, it should be changed to “coordinates”. The first sentence should probably be rephrased.

 Section 2.3: An more detailed explanation of what the ‘decode_coords=”all”’ argument does could be included.

 Section 2.3, last paragraph: ‘in- formation’ -> ‘information’

 Section 2.3, last paragraph: ‘The methods presented here work consistently regardless of the presence or absence of this information’. It is unclear what do ‘methods’ and ‘information’ refer to here.

 Section 3, paragraph 1: ‘which are commonly assumed to be EPSG:4326’ How does one know that this can be assumed? What if it cannot?

 Section 3 paragraph 1: ‘On rare occasions, this information is encoded only in the first time step’ What should be done in this case?

 Conclusion: the readability of this section could benefit from dividing it into 2-3 paragraphs.

Is the rationale for developing the new method (or application) clearly explained?

Yes

Is the description of the method technically sound?

Yes

Are the conclusions about the method and its performance adequately supported by the findings presented in the article?

Yes

If any results are presented, are all the source data underlying the results available to ensure full reproducibility?

Yes

Are sufficient details provided to allow replication of the method development and its use by others?

Yes

Reviewer Expertise:

Meteorology, climate science

We confirm that we have read this submission and believe that we have an appropriate level of expertise to confirm that it is of an acceptable scientific standard.

# Reviewer report: Reviewer response for version 2

**DOI:** 10.21956/openreseurope.23914.r67891

**Published:** 2026-01-29

**Authors:** David Bromwich

**Affiliations:** 1 The Ohio State University, Columbus, Ohio, USA

The authors provide two approaches to making Regional Climate Model output easily usable in GIS with full use of Python libraries. Problems that can be encountered with the Coordinate Reference System are the main consideration. Polar climate simulations from HCLIM and MAR are used to show the effects of transformations. I have nothing to add to the extensive prior reviews.

Is the rationale for developing the new method (or application) clearly explained?

Yes

Is the description of the method technically sound?

Yes

Are the conclusions about the method and its performance adequately supported by the findings presented in the article?

Yes

If any results are presented, are all the source data underlying the results available to ensure full reproducibility?

Yes

Are sufficient details provided to allow replication of the method development and its use by others?

Yes

Reviewer Expertise:

Polar atmospheric modeling using NetCDF output.

I confirm that I have read this submission and believe that I have an appropriate level of expertise to confirm that it is of an acceptable scientific standard.

# Reviewer report: Reviewer response for version 2

**DOI:** 10.21956/openreseurope.23914.r67435

**Published:** 2026-01-28

**Authors:** Aleksi Nummelin

**Affiliations:** 1 Finnish Meteorological Institute,, Helsinki, Finland

This is my first review of a manuscript by Cherblanc et al. titled ‘On solving coordinate problems in climate model output and other geospatial datasets’. This paper introduces a new set of methods to make regional climate model output more accessible for scientist to use. In particular, they suggest methods that allow using the data with and without remapping it.

 I think the manuscript makes a good job in what it aims to do. I also realize that the manuscript has already been revised, but I would still ask the authors to consider the following, after which I would recommend the manuscript for indexing.

 1) I would suggest referring to xESMF ( https://xesmf.readthedocs.io/en/stable/) and perhaps also https://gcm-filters.readthedocs.io/en/latest/ . These tools originate from the oceanographic community but could be beneficial for the audience of this paper. The manuscript provides a reference to the Pangeo community, which xESMF is technically part of, but I think it would be worth referencing this package directly as it very closely related to what the authors are aiming to do. Here it might be worth noting that while the authors focus on CRS as the basis for projecting data, xESMF just uses longitude, latitude information. For example, your example 4.1 could be (more) easily and efficiently done with xESMF as the data is on a grid. The griddata-function always works, but since the data is on a grid, I believe the BivariateSpline ( https://docs.scipy.org/doc/scipy-1.17.0/reference/generated/scipy.interpolate.BivariateSpline.html#scipy.interpolate.BivariateSpline) based methods would be more appropriate.

 2) Section 7 makes the point that CDO is faster than any of the python based approaches. However, xESMF allows saving the remapping weights to a file similar to CDO, and I believe it is actually a bit faster than CDO. I would encourage the authors to try it out.

 3) The manuscript makes no mention of staggered grids. Especially oceanographic models, and I believe some regional atmospheric models, use a staggered grids i.e. grids in which velocity and tracer grids are not the same (probably most commonly variants of Arakawa-c grid). It could be useful to mention this point in discussion section, for example saying that care needs to be taken when working with raw model output on staggered grids.

Is the rationale for developing the new method (or application) clearly explained?

Yes

Is the description of the method technically sound?

Partly

Are the conclusions about the method and its performance adequately supported by the findings presented in the article?

Partly

If any results are presented, are all the source data underlying the results available to ensure full reproducibility?

Yes

Are sufficient details provided to allow replication of the method development and its use by others?

Yes

Reviewer Expertise:

Earth System Modelling

I confirm that I have read this submission and believe that I have an appropriate level of expertise to confirm that it is of an acceptable scientific standard.

# Reviewer report: Reviewer response for version 2

**DOI:** 10.21956/openreseurope.23914.r67886

**Published:** 2026-01-14

**Authors:** Alvaro Escribano

**Affiliations:** 1 Universidad Carlos III de Madrid,, Madrid, Madrid, Spain

This paper provides useful computer programs (post-processing methods) for people working with data from regional climate models (RCM). Sometimes, these data sets of grids have coordinate problems related to geospatial references, especially in the Arctic and Antarctic, due to rotations of longitude and latitude grids. They present ways to read coordinates correctly from different coordinate reference systems.

 I am not an expert in coordinate systems, and therefore, I do not think I can add any other comment on top of the two referee reports available. However, as an analyst of data from the Arctic and Antarctic, I think they address an important issue.

Is the rationale for developing the new method (or application) clearly explained?

Yes

Is the description of the method technically sound?

Yes

Are the conclusions about the method and its performance adequately supported by the findings presented in the article?

Yes

If any results are presented, are all the source data underlying the results available to ensure full reproducibility?

Yes

Are sufficient details provided to allow replication of the method development and its use by others?

Yes

Reviewer Expertise:

I have analysed data on the volume of ice from the Arctic and Antarctic

I confirm that I have read this submission and believe that I have an appropriate level of expertise to confirm that it is of an acceptable scientific standard.

# Reviewer report: Reviewer response for version 2

**DOI:** 10.21956/openreseurope.23914.r66189

**Published:** 2026-01-05

**Authors:** Srinidhi Gadde

**Affiliations:** 1 Universiteit Twente, Enschede, Enschede,, The Netherlands

The Authors have clarified all my questions and updated the manuscript. The manuscript is fit for indexing.

Is the rationale for developing the new method (or application) clearly explained?

Partly

Is the description of the method technically sound?

Yes

Are the conclusions about the method and its performance adequately supported by the findings presented in the article?

Partly

If any results are presented, are all the source data underlying the results available to ensure full reproducibility?

Yes

Are sufficient details provided to allow replication of the method development and its use by others?

Yes

Reviewer Expertise:

Climate modelling and atmospheric observations.

I confirm that I have read this submission and believe that I have an appropriate level of expertise to confirm that it is of an acceptable scientific standard.

# Reviewer report: Reviewer response for version 2

**DOI:** 10.21956/openreseurope.23914.r66190

**Published:** 2026-01-02

**Authors:** Deniz Bozkurt

**Affiliations:** 1 University of Valparaíso, Valparaíso, Valparaíso Region, Chile

I have reviewed the revised version of the manuscript and am satisfied that the authors have addressed the reviewers’ comments appropriately. I appreciate the careful revisions and would like to thank the authors for their constructive responses.

 Based on these changes, I am happy to amend my previous status to Approved.

Is the rationale for developing the new method (or application) clearly explained?

Yes

Is the description of the method technically sound?

Yes

Are the conclusions about the method and its performance adequately supported by the findings presented in the article?

Partly

If any results are presented, are all the source data underlying the results available to ensure full reproducibility?

Yes

Are sufficient details provided to allow replication of the method development and its use by others?

Yes

Reviewer Expertise:

Regional climate modeling, climate dynamics, climate change

I confirm that I have read this submission and believe that I have an appropriate level of expertise to confirm that it is of an acceptable scientific standard.

# Reviewer report: Reviewer response for version 1

**DOI:** 10.21956/openreseurope.22147.r61902

**Published:** 2025-11-08

**Authors:** Deniz Bozkurt

**Affiliations:** 1 University of Valparaíso, Valparaíso, Valparaíso Region, Chile

This is a very useful and clearly written paper that addresses a practical issue faced by many people working with regional climate model (RCM) data, namely, problems with coordinate reference systems and inconsistent metadata. The examples are concrete and the explanations are generally accessible. That said, a few minor points need clarification and polishing before publication. The comments below highlight where the paper could be clearer and more accurate.

 1. The introduction and abstract seem to mix two things: dealing with older, non–CF-compliant model outputs and giving general guidance for modern users. It would help a lot if you stated early on whether the paper mainly targets legacy or non-standard datasets or if the problem is still frequent in current workflows.

 2. The paper sometimes reads as if new algorithms are proposed, while in practice it is a practical combination of existing tools (xarray, rioxarray, CDO, etc.). Therefore, maybe better to phrase it more as an application or *best-practice guide* rather than a “new method”.

 3. The conclusion lists two approaches but doesn’t really help a reader choose between them. A short paragraph (or even a small table) summarizing when to use coordinate-only reprojection vs. full interpolation/regridding would make this much more practical.

 4. In some places it is said that the CRS is “missing,” yet the Python output shows that it can be recovered using decode_coords="all". That might confuse readers. Please either use one case where CRS is truly missing or clearly say that the example *simulates* a missing CRS to demonstrate the method.

 5. The transformations assume generic EPSG:3031 or 4326, which might not match each model’s own parameters. A short caution note about verifying model-specific projection parameters would make this section more robust.

 6. Since rioxarray.rio.reproject_match() already performs reprojection if CRS is defined, it would be helpful to mention that users should first try built-in decoding before manually transforming coordinates.

 7. The text says CDO is faster, but that’s not shown. You could simply add one sentence like: “In our tests on a standard workstation, CDO was roughly X times faster for large batches of files.” 

 8. You use cubic interpolation but don’t explain why. A short note on why you chose “cubic” (e.g. smoother fields, continuous derivatives) or when users might prefer “bilinear” or “nearest” would help readers make informed choices.

 9. The discussion could benefit from a bit more about how large the differences between methods actually are. Even a qualitative statement (“visual differences were small except near sharp gradients”) would round out the comparison.

 10. References 11 and 17 appear to be duplicates (HCLIM38).

 11. Page 7, “ForCDO” → “For CDO”.

 12. Some figures (especially Figs. 2–5) have minimal or unclear axis labels (e.g., “X”, “Y” without units). I think it would be better to add clearer captions and labeling, including units, color bars, and projection type, to make the figures easier to interpret.

Is the rationale for developing the new method (or application) clearly explained?

Yes

Is the description of the method technically sound?

Yes

Are the conclusions about the method and its performance adequately supported by the findings presented in the article?

Partly

If any results are presented, are all the source data underlying the results available to ensure full reproducibility?

Yes

Are sufficient details provided to allow replication of the method development and its use by others?

Yes

Reviewer Expertise:

Regional climate modeling, climate dynamics, climate change

I confirm that I have read this submission and believe that I have an appropriate level of expertise to confirm that it is of an acceptable scientific standard, however I have significant reservations, as outlined above.

# Response

**Published:** 2025-12-01

**Authors:** Clément Cherblanc

**Affiliations:** PICE, Niels Bohr Institute, Copenhagen, Capital Region of Denmark, Denmark

Response to Deniz Bozkurt.

 Thank you for your detailed and thoughtful comments on our original submission. We believe that addressing your review has substantially improved the manuscript. We have copy-pasted your points here in black, and responded to them using purple.  

**Comment: **1. The introduction and abstract seem to mix two things: dealing with older, non–CF-compliant model outputs and giving general guidance for modern users. It would help a lot if you stated early on whether the paper mainly targets legacy or non-standard datasets or if the problem is still frequent in current workflows.  

**Response: **We introduced a clearer limitation with legacy, non CF-compliant, and technical limitations to reprojection. We added a sentence clarifying the dual-goal of education and providing examples to connect datasets that are not compatible out-of-the-box. In our experience users are often working with ad-hoc produced datasets, that have not necessarily been CMORised and this paper aims specifically to address these. We have examples of modern dataset that are not CF-compliants, for example gridMET, but we are not pointing fingers and therefore do not report them in the main paper.  

**Comment: **2. The paper sometimes reads as if new algorithms are proposed, while in practice it is a practical combination of existing tools (xarray, rioxarray, CDO, etc.). Therefore, maybe better to phrase it more as an application or best-practice guide rather than a “new method”.  

**Response: **Agreed. In the problem onset section, the last paragraph of introduction, we replaced “methods” by “best-practices” to clarify this.  

**​​​​​​​Comment: **3. The conclusion lists two approaches but doesn’t really help a reader choose between them. A short paragraph (or even a small table) summarizing when to use coordinate-only reprojection vs. full interpolation/regridding would make this much more practical.  

**​​​​​​​Response: **This is a good point, it is implicit that both techniques have their strengths and weaknesses, so we have strengthened this point in the conclusion as suggested to outline when we would suggest using each method, in particular as regridding and interpolating datasets as suggested here may introduce artefacts.  

**​​​​​​​Comment: **4. In some places it is said that the CRS is “missing,” yet the Python output shows that it can be recovered using decode_coords="all". That might confuse readers. Please either use one case where CRS is truly missing or clearly say that the example simulates a missing CRS to demonstrate the method.  

**​​​​​​​Response: **I have fixed an occurrence in Section 3. We did not find another occurrence.  

**​​​​​​​Comment: **5. The transformations assume generic EPSG:3031 or 4326, which might not match each model’s own parameters. A short caution note about verifying model-specific projection parameters would make this section more robust.  

**​​​​​​​Response: **Thankyou for this suggestion,  we did not give a proper introduction to the EPSG number and we have now introduced this concept more carefully and given advice on how to pick the appropriate CRS.  

**​​​​​​​Comment: **6. Since rioxarray.rio.reproject_match() already performs reprojection if CRS is defined, it would be helpful to mention that users should first try built-in decoding before manually transforming coordinates.  

**​​​​​​​Response: **​​​​​​​Indeed, we made it clear at the end of section 2 and in section 3. The default rio.reproject function will reproject the dataset to a regular grid, which implies reprojection biases, especially near the poles. We added a small introduction in section 3 to strengthen the use of coordinate-only reprojection.  

**​​​​​​​Comment: **7. The text says CDO is faster, but that’s not shown. You could simply add one sentence like: “In our tests on a standard workstation, CDO was roughly X times faster for large batches of files.”   

**​​​​​​​Response: **We reported numbers in the section, showing how CDO reprojection scales up by opposition to Python’s, this is now made more explicit in the text.  

**​​​​​​​Comment: **8. You use cubic interpolation but don’t explain why. A short note on why you chose “cubic” (e.g. smoother fields, continuous derivatives) or when users might prefer “bilinear” or “nearest” would help readers make informed choices.  

**​​​​​​​Response: **Discussing the interpolation method is beyond the scope of this paper but we have added a line to state that we use this method for simplicity but other interpolation choices might be more relevant in different cases.   

**​​​​​​​Comment: **9. The discussion could benefit from a bit more about how large the differences between methods actually are. Even a qualitative statement (“visual differences were small except near sharp gradients”) would round out the comparison.  

**​​​​​​​Response: **If you are talking about the difference between CDO and Python, using the same source, target and method, they are non-existent. If you are talking about the difference on Figure 3, it is only meant as an example of comparing the output of two different models, now that they exist on the same grid. They did not before. We have added a couple of lines in the figure caption to clarify this.  

**​​​​​​​Comment: **10. References 11 and 17 appear to be duplicates (HCLIM38).  

**​​​​​​​Response: **Thank you for noticing this. We thought that we fixed this during the typesetting process with the ORE editor, but it seems that this was not fixed until now!  

**​​​​​​​Comment: **11. Page 7, “ForCDO” → “For CDO”.  

**​​​​​​​Response: **Good catch! Fixed.  

**​​​​​​​Comment: **12. Some figures (especially Figs. 2–5) have minimal or unclear axis labels (e.g., “X”, “Y” without units). I think it would be better to add clearer captions and labeling, including units, color bars, and projection type, to make the figures easier to interpret.  

**​​​​​​​Response: **​​​​​​​We purposely left these as minimalist as possible. The code to make figures 1, 2, 4 (and 5 to some extent) is in the paper main body, we want to show critical differences both with code, for practical purposes, and with the exact output in the paper. We want the reader to immediately understand the difference between the plots, or their purpose, with no lines of code that are essentially cosmetic. We believe that the captions should be left as is.

# Reviewer report: Reviewer response for version 1

**DOI:** 10.21956/openreseurope.22147.r60626

**Published:** 2025-10-01

**Authors:** Srinidhi Gadde

**Affiliations:** 1 Universiteit Twente, Enschede, Enschede,, The Netherlands

Review of “On solving coordinate problems in climate model output and other geospatial datasets”

**Summary**

 This paper presents two practi cal approaches for handling Regional Climate Model (RCM) output where coordinate reference system (CRS) metadata may be incomplete, absent, or inconsistent. The authors demonstrate:

1. Reprojecting only the coordinates using pyproj, which avoids altering the data
2. Reprojecting the entire dataset onto a regular grid using interpolations, with Python or CDO.

The work is framed as a practical guide for non-specialists, supported by open-source tools and reproducible code.  The paper, addresses a real barrier for non-specialists working with RCM outputs, particularly in polar regions with rotated/polar grids. Clearly demonstrates two complimentary workflows, with discussion of their trade-offs.

 This is a useful and timely paper that will benefit non-specialists who need to handle model outputs with incomplete or inconsistent metadata. I recommend indexing the paper with revisions, focusing on clarifying the prevalence of the problem, why were the  built-in mechanisms in the Python packages not used, and clarifying the prevalence of the non-compliance to CF-standard in the climate model outputs. See below for detailed comments.

**Major comments**

 My major comment is that the paper motivates its workflows by noting that RCM outputs often lack CRS information or use non-standard conventions. However, most modern climate datasets are CF-compliant and therefore must include grid_mapping information. This raises the question of how widespread the “missing CRS” problem still is in practice. It would help the readers, if the authors should clarify:

1. Whether they are targeting mostly legacy or non-standard datasets
2. How common such cases remain compared to fully CF-compliant datasets?

Providing concrete examples would strengthen the justification for the workflows and help readers understand when they are most needed.

 Related comments

1. Both example datasets used in the paper already contain CF-compliant grid_mapping metadata with full projection information. Standard tools (decode_coords=”all” in xarray, or CRS.from_cf() in pyproj) can read this directly. Presenting them as if CRS were absent may confuse non-specialists. I recommend either (a) adding an example where CRS is genuinely missing, or (b) explicitly clarifying that CRS metadata is available but the workflows are shown for general cases where it might be incomplete or corrupted
2. The workflows manually assign EPSG codes (3031/3572 and soon) and transform the coordinates. Wihle workable, these assumptions may not match model-specific parameters encoded in NetCDF metadata. The authors should explain why they did not use built-in mechanisms (decode_coords=”all”, rioxarray.rio.write_crs, CRS.from_cf()) and note the risks of assuming generic EPSG codes.  If the paper is meant as a guide for non-specialists then perhaps a comment saying that built-in mechanism such as decode_coords should be used before using the transformation would make it beneficial for the non-experts.
3. The conclusion contrasts the two workflows. But the decision criteria could be made clearer. A short guide (for example, use reprojection for visualization purposes without altering values, use interpolation/regridding for model intercomparison) would make the paper more practical. 

Is the rationale for developing the new method (or application) clearly explained?

Partly

Is the description of the method technically sound?

Yes

Are the conclusions about the method and its performance adequately supported by the findings presented in the article?

Partly

If any results are presented, are all the source data underlying the results available to ensure full reproducibility?

Yes

Are sufficient details provided to allow replication of the method development and its use by others?

Yes

Reviewer Expertise:

Climate modelling and atmospheric observations.

I confirm that I have read this submission and believe that I have an appropriate level of expertise to confirm that it is of an acceptable scientific standard, however I have significant reservations, as outlined above.

# Response

**Published:** 2025-12-01

**Authors:** Clément Cherblanc

**Affiliations:** PICE, Niels Bohr Institute, Copenhagen, Capital Region of Denmark, Denmark

Response to Srinidhi Gadde.

 Thank you for your comments on our original submission. They did raise important questions and resulted in an improvement of the manuscripts. We have copy-pasted your points here in black and responded to them using purple.  

**Comment: **My major comment is that the paper motivates its workflows by noting that RCM outputs often lack CRS information or use non-standard conventions. However, most modern climate datasets are CF-compliant and therefore must include grid_mapping information. This raises the question of how widespread the “missing CRS” problem still is in practice. It would help the readers, if the authors should clarify:

1. Whether they are targeting mostly legacy or non-standard datasets
2. How common such cases remain compared to fully CF-compliant datasets?

Providing concrete examples would strengthen the justification for the workflows and help readers understand when they are most needed.  

**Response:** You are correct that a majority of recent, user-ready, output are aimed to be CF-compliant. In practice, there are legacy issues, and not all datasets are CF-compliant (gridMET is an example of a popular dataset with an invalid geotransform, which had to be reprojected with the methods presented in this paper, we do not aim at pointing fingers and do not report it in the paper). Other users of climate model outputs are often working with ad hoc outputs that have not necessarily been through full post-processing. A restricted use of a dataset only because it is not compliant to the CF convention would be very unfortunate. In addition, errors are more likely for high-latitude datasets, a better understanding of the processes is needed, otherwise causing large errors, especially with automatic reprojection. We included those items in the introduction of the paper, to strengthen our motivations, though. 

**Comment:**

1. Both example datasets used in the paper already contain CF-compliant grid_mapping metadata with full projection information. Standard tools (decode_coords=”all” in xarray, or CRS.from_cf() in pyproj) can read this directly. Presenting them as if CRS were absent may confuse non-specialists. I recommend either (a) adding an example where CRS is genuinely missing, or (b) explicitly clarifying that CRS metadata is available but the workflows are shown for general cases where it might be incomplete or corrupted

**Response:** The last part of 2.3 specifically shows that decode_coords works in that case. We added a sentence to emphasize that our code will work with either case, and that using the built-in functionality may bring other issues (ie: regular-grid reprojection that introduces biases). Section 5 now also benefits from a clarification: that built-in functions will work, but offer limited control of the target grid properties.  

**Comment:**

1. The workflows manually assign EPSG codes (3031/3572 and soon) and transform the coordinates. Wihle workable, these assumptions may not match model-specific parameters encoded in NetCDF metadata. The authors should explain why they did not use built-in mechanisms (decode_coords=”all”, rioxarray.rio.write_crs, CRS.from_cf()) and note the risks of assuming generic EPSG codes.  If the paper is meant as a guide for non-specialists then perhaps a comment saying that built-in mechanism such as decode_coords should be used before using the transformation would make it beneficial for the non-experts.

**Response:** ​​​​​​​You are correct that we did not document the choice of this CRS, and this choice may appear obscure to an inexperienced reader. I have added several sentences in section 3 giving background for that choice, given that this CRS is commonly used in our field. If no recommendation is available, and there is no standard for a region, a UTM should be used.
